# Electroacupuncture at Quchi and Zusanli treats cerebral ischemia-reperfusion injury through activation of ERK signaling

**DOI:** 10.3892/etm.2013.1030

**Published:** 2013-03-26

**Authors:** GUANLI XIE, SHANLI YANG, AZHEN CHEN, LAN LAN, ZHICHENG LIN, YANLIN GAO, JIA HUANG, JIUMAO LIN, JUN PENG, JING TAO, LIDIAN CHEN

**Affiliations:** 1 College of Rehabilitation Medicine, Fujian University of Traditional Chinese Medicine, Fuzhou, Fujian 350108, P.R. China; 2 MOE Key Laboratory of Traditional Chinese Medicine on Osteology and Traumatology and Exercise Rehabilitation, Fujian University of Traditional Chinese Medicine, Fuzhou, Fujian 350108, P.R. China; 3 Fujian Key Laboratory of Exercise Rehabilitation, Fujian University of Traditional Chinese Medicine, Fuzhou, Fujian 350108, P.R. China; 4 Fujian Key Laboratory of Integrative Medicine on Geriatrics, Fujian University of Traditional Chinese Medicine, Fuzhou, Fujian 350108, P.R. China

**Keywords:** electroacupuncture, Quchi (LI11) and Zusanli (ST36) acupoints, cerebral ischemia/reperfusion, extracellular signal-regulated kinase pathway, cell proliferation

## Abstract

The extracellular signal-regulated kinase (ERK) pathway, a critical mediator of cell proliferation, is activated in cerebral ischemia/reperfusion (I/R) injury and is therefore a key target in the treatment of ischemic stroke. Acupuncture has long been used in China to clinically treat stroke. However, the precise mechanism of its neuroprotective activities remains largely unknown. In the present study, a focal cerebral I/R-injured rat model was used to evaluate the *in vivo* therapeutic efficacy of electroacupuncture (EA) and investigate the underlying molecular mechanisms. EA significantly ameliorated neurological deficits and cerebral infarction in cerebral I/R-injured rats. Moreover, EA significantly increased the phosphorylation levels of ERK, as well as the protein expression levels of Ras, cyclin D1 and cyclin-dependent kinase (CDK)4. Consequently, EA-mediated activation of the ERK pathway resulted in the stimulation of cerebral cell proliferation. The present data suggest that EA at the Quchi and Zusanli acupoints exerts a neuroprotective effect in ischemic stroke via the activation of ERK signaling.

## Introduction

Ischemic stroke, a commonly encountered and frequently occurring clinical disease, is a complication of hypertension, heart disease and diabetes mellitus (1), which occurs when the blood supply to a part of brain is interrupted or severely reduced, resulting in oxygen and nutrient deprivation in brain tissues (2). Acupuncture, a medicinal methodology originating in ancient China, has long been used as a complementary and alternative therapy in a number of East Asian countries as well as more recently in Western society (3). The clinical efficacy of acupuncture in stroke rehabilitation has been demonstrated in numerous studies (4–9) and the Zusanli (ST36) and Quchi (LI11) acupoints are the acupoints most commonly used to clinically treat stroke in China (10,11). However, the precise mechanism of the neuroprotective effect remains to be elucidated.

The pathogenic mechanisms of ischemic stroke are complex. During ischemia/reperfusion (I/R) injury, cells undergo rapid changes which lead to perturbations in signaling pathways, resulting in an imbalance between cell proliferation and apoptosis (12,13). Extracellular signal-regulated kinase (ERK) signaling is one of the major cell-survival and proliferation pathways. As a major subfamily of the mitogen-activated protein kinases (MAPKs), the activation of ERKs is regulated by a central three-tiered kinase core consisting of a MAPK kinase kinase (e.g., Raf), MAPK kinase (e.g., MEK) and MAPK, wherein Raf phosphorylates MEK which in turn phosphorylates and activates the ERK (14). By altering the levels and activities of transcription factors, the activation of the ERK pathway regulates the expression of various cell cycle-regulatory genes, including cyclin D1 and cyclin-dependent kinase (CDK)4, thus mediating the promotion of cell proliferation (15,16). The role of the ERK pathway in cerebral I/R injury has been studied intensively (17–19), suggesting that the activation of ERK signaling is a promising target for stroke treatment.

In present study, a focal cerebral I/R-injured rat model was used to elucidate the neuroprotective mechanism of electroacupuncture (EA) at the Quchi and Zusanli acupoints, evaluate the therapeutic efficacy of EA against ischemic stroke and investigate its effect on the ERK pathway.

## Materials and methods

### Materials and reagents

Ras, ERK1/2, phospho-p44/42 (Thr^202^/Thr^204^), cyclin D1 and horseradish peroxidase (HRP)-conjugated secondary antibodies were obtained from Cell Signaling Technology, Inc. (Beverly, MA, USA). Mouse proliferating cell nuclear antigen (PCNA) immunohistochemical (IHC) kits were purchased from Beijing Golden Bridge Biotechnology Co., Ltd. (Beijing, China), while rat CDK4 antibody was obtained from Abcam (Cambridge, MA, USA). All other chemicals, unless stated otherwise, were obtained from Sigma-Aldrich (St. Louis, MO, USA).

### Animals

Male Sprague-Dawley rats (initial body weights ∼250 g) were obtained from Shanghai SLAC Laboratory Animal Co., Ltd. (Shanghai, China) and housed under pathogen-free conditions with a 12 h light/dark cycle. Food and water were provided *ad libitum* throughout the experiment. All animal treatments were strictly in accordance with the international ethical guidelines and National Institutes of Health guide concerning the Care and Use of Laboratory Animals. The study was approved by the Institutional Animal Care and Use Committee of Fujian University of Traditional Chinese Medicine (Fuzhou, China).

### Establishment of the cerebral I/R-injured rat model and animal groups

The I/R-injured model was established by middle cerebral artery (MCA) occlusion (MCAO) as described previously (20). Briefly, after each rat was anesthetized by intraperitoneal injection of 10% chloral hydrate (300 mg/kg), the left common carotid artery (CCA), left external carotid artery (ECA) and internal carotid artery (ICA) were carefully exposed via a midline neck incision. The left MCA was occluded by introducing an embolus through the ICA. The CCA and the ECA were permanently blocked. Focal cerebral ischemia was induced by occluding the left common carotid artery (MCA) when the tip of catheter reached the origin of MCA (18–22 mm). Reperfusion was achieved by removing the thread after 2 h of occlusion to restore the blood supply to the MCA area. Heat preservation was considered throughout the process. The rectal temperatures of the rats were maintained at 37°C throughout the surgical procedures. Sham-operated control (SC) animals underwent the same surgical procedure, but no arterial occlusion was performed and no embolus used.

The animals were randomly divided into 3 groups (n=8) as follows: i) in the SC group, the rats underwent neck dissection and the exposure of the blood vessels, but no arterial occlusion; ii) in the ischemic control (IC) group, the left MCA was blocked for 2 h and then recanalized, iii) in the EA group, the surgical procedure was same as that in the IC group. After recovery from the I/R surgery and 2 h of reperfusion, EA treatment was performed daily for 30 min. Acupuncture needles (0.3 mm diameter) were inserted 2–3 mm deep into the Quchi (LI11) and Zusanli (ST36) acupoints on the right paralyzed limb. Stimulation was then generated with the EA apparatus (Model G6805; SMIF, Shanghai, China) and the stimulation parameters were set as disperse waves of 1 and 20 Hz.

### Evaluation of neurological deficit scores

At 2 or 24 h after I/R, the neurological deficit score was examined in a blinded manner as described previously (20): a score of 0 indicated no neurological deficits; 1 (failure to fully extend right forepaw) indicated mild focal neurological deficits; 2 (circling to the right) and 3 (falling to the right) indicated moderate focal neurological deficits; rats with a score of 4 were not able to walk independently and exhibited a depressed level of consciousness. Mice that scored 0 or 4 were eliminated from the experiment.

### Measurement of cerebral infarct volume

After cerebral I/R injury for 24 h, the rats were anesthetized with 10% chloral hydrate by intraperitoneal injection. Each rat was perfused transcardially with 0.9% NaCl and the brain was removed. The brain was sectioned in the coronal plane into 2-mm thick slices. The slices were placed in 2% 2,3,5-triphenyltetrazolium chloride (TTC) in phosphate-buffered saline (PBS) at 37°C for 20 min and fixed by immersion in 4% buffered formaldehyde solution (21). The normal area of the brain was stained dark red based on intact mitochondrial function, whereas the infarct area remained unstained. Each brain slice was scanned with a high-resolution digital camera (Canon SX20; Canon Inc., Tokyo, Japan) and the infarct was quantified as a percentage of the total brain volume using a Motic 6.0 system (Motic, Xiamen, China).

### Immunohistochemistry of PCNA, cyclin D1 and CDK4

Each rat was anesthetized and perfused transcardially with 0.9% NaCl and 4% paraformaldehyde through the left ventricle and the brain was removed. Samples were fixed in cold 4% paraformaldehyde and processed into 5-*μ*m thick sections. PCNA, cyclin D1 and CDK4 levels were analyzed with an immunohistochemistry assay kit (DS-005; Beijing Golden Bridge Biotechnology Co., Ltd.) according to the manufacturer’s instructions. For staining, the slides were placed in 3% hydrogen peroxide and normal serum for 10 min at 37°C, to block nonspecific protein activity. This was followed by an incubation at 4°C overnight with the primary antibodies, rabbit anti-CDK4 (1:50; Abcam), rabbit anti-cyclin D1 (1:400; Cell Signaling Technology, Inc.) and mouse anti-PCNA (Beijing Golden Bridge Biotechnology Co., Ltd.). After incubation with primary antibodies, the sections were washed three times in PBS and incubated with the secondary antibodies. The brain sections were stained with alkaline phosphatase (AP)-red or diaminobenzidine (DAB) staining solutions. PCNA-positive cells were stained red, while cyclin D1 and CDK4-positive cells were stained sepia. Positive cells were counted in four randomly selected microscopic fields at ×400 magnification. The positive rate was expressed as the ratio of red- or sepia-stained cells.

### Western blotting analysis

Ischemic cerebral tissues were homogenized in nondenaturing lysis buffer and centrifuged at 12,000 × g for 15 min. The supernatants were collected and frozen at −80°C until immunoblotting. The protein concentration of each homogenate was determined. Equal amounts of protein (50 *μ*g) were loaded onto 12% SDS-PAGE gels for electrophoresis, then transferred to a PVDF membrane. After blocking in 5% non-fat dry milk in 0.1 M Tris-buffered saline (TBS)-0.1% Tween-20 (TBST), the proteins were detected with primary antibodies against Ras, ERK1/2, p-ERK1/2 and β-actin (dilution, 1:1,000). The proteins were incubated overnight with primary antibodies at 4°C, then with appropriate HRP-conjugated secondary antibodies for 50 min. Blots were developed using enhanced chemiluminescence and images were analyzed using a Bio-Image Analysis System (Bio-Rad, Hercules, CA, USA).

### Statistical analysis

All data were processed using SPSS 16.0. Quantitative data were expressed as the mean ± standard deviation. Differences among the three groups were compared using one-way analysis of variance (ANOVA) and Student’s t-tests. P<0.05 was considered to indicate a statistically significant difference.

## Results

### EA treatment at the Zusanli (ST36) and Quchi (LI11) acupoints alleviates neurological deficits in cerebral I/R-injured rats

The neuroprotective effect of EA was first evaluated by measuring the neurological deficit scores. As shown in Table I, all MCAO rats exhibited clear manifestations of neurological deficits compared with rats in the SC group (P<0.05), indicating successful model construction. Although no significant differences were observed between the IC and EA groups in the clinical evaluation before electric stimulation, EA at Zusanli and Quchi was observed to significantly improve the neurological deficits (P<0.05).

### EA treatment at the Quchi and Zusanli acupoints decreases the infarct volume in cerebral I/R-injured rats

**Figure 1:**
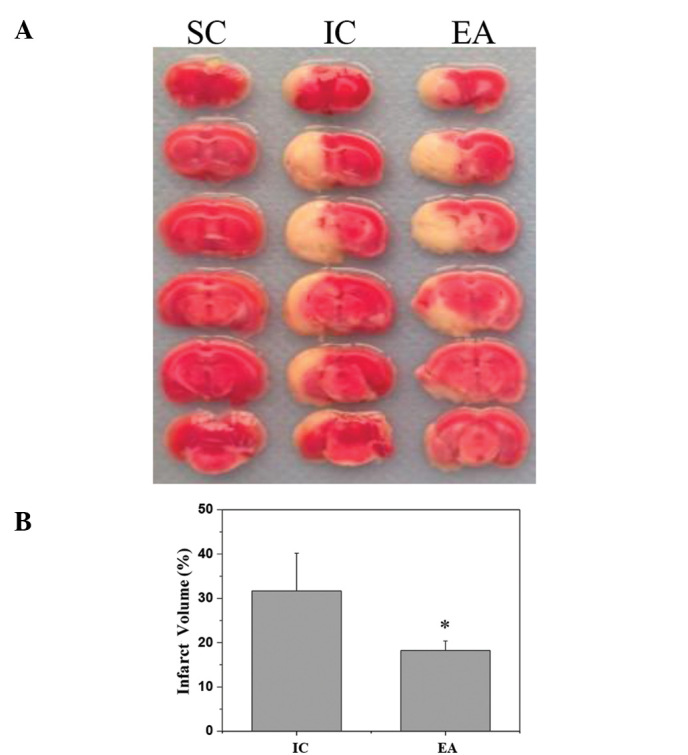
Effect of EA at the Quchi (LI11) and Zusanli (ST36) acupoints on cerebral infarction in I/R injured rats. (A) At the end of the experiment, cerebral tissues from each group were coronally sectioned into 2-mm thick slices and subjected to TTC staining. Images were captured with a high-resolution digital camera. Images are representative of three independent experiments. (B) Infarct volume was quantified as a percentage of the total brain volume using a Motic 6.0 system. Data shown are averages with SE (error bars) from three individual rats in each group. ^*^P<0.05, vs. IC group. I/R, ischemia/reperfusion; SC, sham-operated control; IC, ischemic control; EA, electroacupuncture; TTC, 2,3,5-triphenyltetrazolium chloride.

To further investigate the therapeutic efficacy of EA against cerebral I/R injury, its effect on cerebral infarct volume was evaluated using TTC staining. As shown in Fig. 1, EA at Quchi and Zusanli significantly reduced the cerebral infarct volumes. The total infarct volumes were 31.66±8.53 and 18.25±2.11% of the total brain volume in the IC and EA groups, respectively (P<0.05).

### EA at the Quchi and Zusanli acupoints activates the ERK pathway in cerebral I/R-injured rats

**Figure 2:**
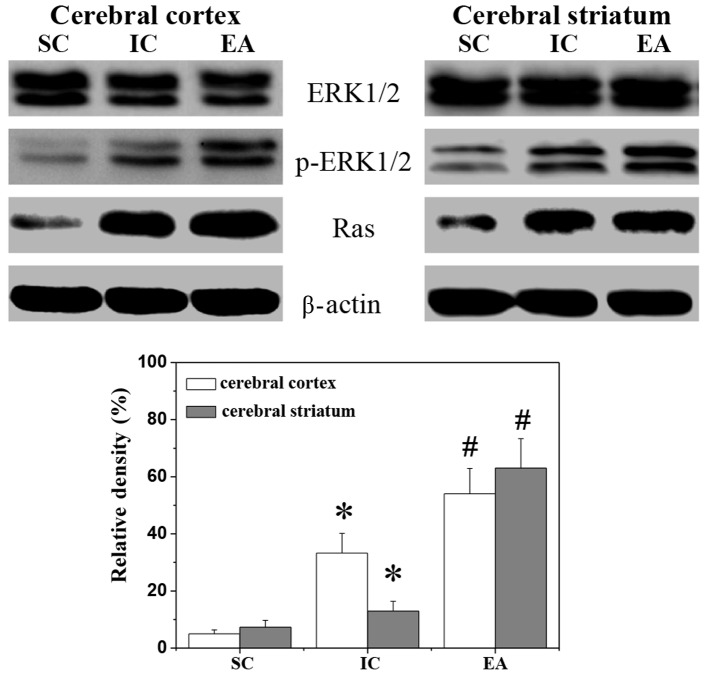
Effect of EA on the ERK1/2 pathway in cerebral I/R-injured rats. The levels of ERK1/2 protein expression and ERK1/2 phosphorylation in the ischemic cerebral cortex and cerebral striatum were determined by western blotting. β-actin was used as the internal control. Relative density was expressed as the optical density of p-ERK1/2 relative to that of tERK1/2. Data are representative of five individual rats in each group. Data are averages with SE (error bars). ^*^P<0.05, vs. SC group; ^#^P<0.05, vs. IC group. I/R, ischemia/reperfusion SC, sham-operated control; IC, ischemic control; EA, electroacupuncture; ERK, extracellular signal-regulated kinase.

To investigate the effect of EA on the ERK pathway, western blotting was performed to examine the expression of Ras and the phosphorylation of ERK in the ischemic cerebral cortex and striatum. As shown in Fig. 2, I/R injury increased the Ras protein expression and the phosphorylation level of ERK. This was consistent with previous studies of the transient focal ischemia model which showed that ERK was activated following I/R and persisted for 24 h (22,23). EA at Zusanli and Quchi further upregulated the protein expression of Ras, as well as ERK phosphorylation, whereas the levels of nonphosphorylated ERK remained unchanged in all three animal groups.

### Electroacupuncture at the Quchi and Zusanli acupoints promotes cell proliferation in cerebral I/R-injured rats

**Figure 3:**
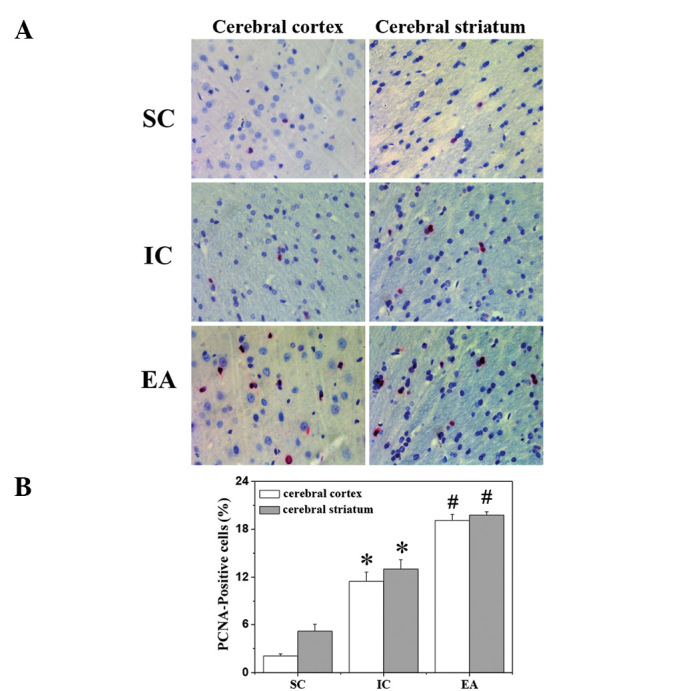
Effect of EA on PCNA-positive cells in cerebral I/R-injured rats. Cerebral tissues from each group (n=5) were processed for an immunohistochemical assay. The nuclei of all cells were visualized by hematoxylin staining and the PCNA-positive cells were stained red with AP-red solution. The PCNA-positive cells were counted at four randomly selected microscopic fields at ×400 magnification. PCNA-positive cell rate was expressed as the ratio of red-stained cells to the blue-stained total cells. Data are averages with SE (error bars). ^*^P<0.05, vs. SC group; ^#^P<0.05, vs. IC group. SC, sham-operated control; IC, ischemic control; EA, electroacupuncture; I/R, ischemia/reperfusion; PCNA, proliferating cell nuclear antigen; AP, alkaline phosphatase.

ERK activation is important in cell proliferation and therefore, the pro-proliferative activity of EA was investigated using IHC staining for PCNA. As shown in Fig. 3, I/R injury increased the percentage of PCNA-positive cells in the ischemic cerebral cortex and striatum of the IC rats compared with the SC group. The percentages of PCNA-positive cells in the ischemic cerebral cortex and striatum of the SC rats were 2.00±0.27 and 5.17±1.61%, respectively, while those in the IC group were 11.48±1.15 and 13.01±1.17% (P<0.05). However, EA at Zusanli and Quchi was observed to significantly promote cell proliferation. The PCNA-positive cell rates of the ischemic cerebral cortex and striatum in the EA group were 19.11±0.77 and 19.77±0.42%, respectively (P<0.05, vs. IC group).

### Electroacupuncture at Quchi and Zusanli increases the expression of cyclin D1 and CDK4 in cerebral I/R-injured rats

**Figure 4:**
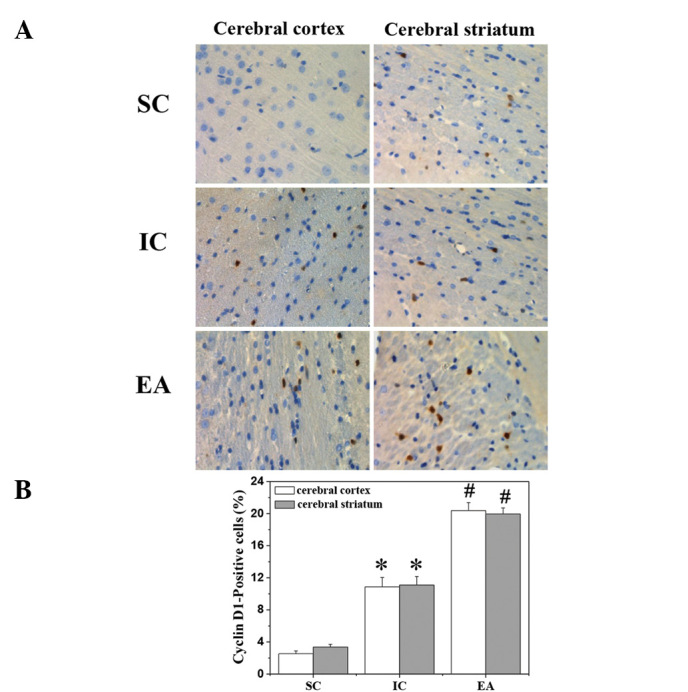
Effect of EA on the cyclin D1-positive cell rate in cerebral I/R-injured rats. Cerebral tissues from each group (n=5) were processed for an immunohistochemical assay. The nuclei of all cells were visualized by hematoxylin staining and the cyclin D1-positive cells were stained sepia with DAB solution. Cyclin D1-positive cells were counted at four randomly selected microscopic fields at ×400 magnification. The cyclin D1-positive cell rate was expressed as the ratio of sepia-stained cells to the blue-stained total cells. Data are averages with SE (error bars). ^*^P<0.05, vs. SC group; ^#^P<0.05, vs. IC group. SC, sham-operated control; IC, ischemic control; EA, electroacu-puncture; I/R, ischemia/reperfusion; DAB, diaminobenzidine.

**Figure 5:**
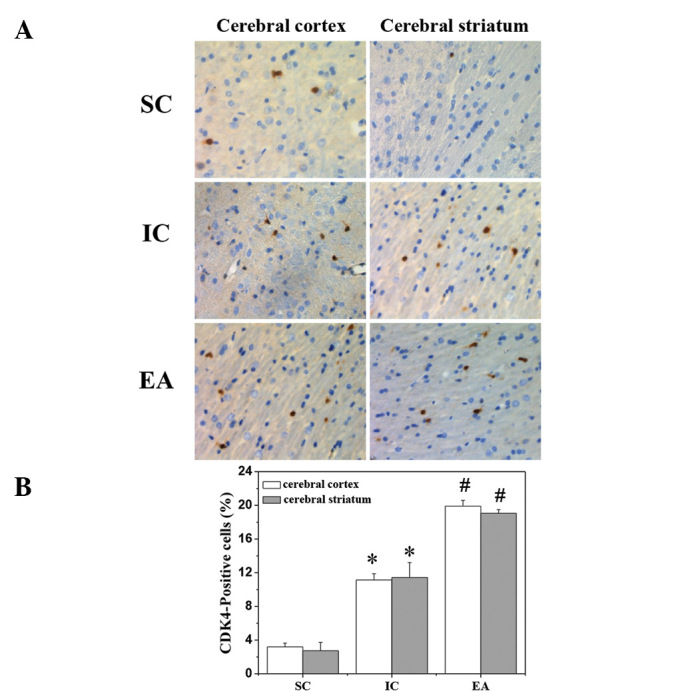
Effect of EA on the CDK4-positive cell rate in cerebral I/R-injured rats. Cerebral tissues from each group (n=5) were processed for an immunohistochemical assay. The nuclei of all cells were visualized by hematoxylin staining and the CDK4-positive cells were stained sepia with DAB solution. CDK4-positive cells were counted at four randomly selected microscopic fields ×400 magnification. The CDK4-positive cell rate was expressed as the ratio of sepia-stained cells to the blue-stained total cells. Data are averages with SE (error bars). ^*^P<0.05, vs. SC group; ^#^P<0.05, vs. IC group. SC, sham-operated control; IC, ischemic control; EA, electroacupuncture; CDK4, cyclin-dependent kinase 4; I/R, ischemia/reperfusion; DAB, diaminobenzidine.

To further investigate the mechanism of the pro-proliferative activity of EA, its effect on the protein expression of cyclin D1 and CDK4 was evaluated using IHC staining (Figs. 4 and 5). Consistent with the previous findings, the protein expression levels of cyclin D1 and CDK4 in the ischemic cerebral cortex and striatum were increased by I/R injury and further upregulated by EA treatment.

## Discussion

The ERK1/2 pathway is a critical mediator of cell proliferation, which is activated in response to growth factors (24,25), oxidative stress (26) and glutamate receptor stimulation (19,27), and promotes progression from the G1 to the S-phase by regulating the cyclin D1-CDK4 complex (15,16,28). Numerous studies have reported that the activation of the ERK1/2 pathway is markedly associated with protection from cerebral I/R injury, decreasing the infarct size and promoting cerebral cell proliferation (29–32). Therefore, promoting cerebral cell proliferation via the activation of ERK signaling is a promising strategy for the treatment of ischemic stroke. Acupuncture is an alternative medicine methodology that has long been used in China to treat various diseases. Previous studies have demonstrated the clinical efficacy of acupuncture in stroke rehabilitation. On the basis of data in the literature, the Zusanli (ST36) and Quchi (LI11) acupoints have commonly been used in China to clinically treat stroke. However, the mode of action of the neuroprotective activities of EA remain poorly understood.

In the present study, a focal cerebral I/R rat model was used to demonstrate that EA at Zusanli and Quchi for only 24 h had a neuroprotective effect as evidenced by improved neurological deficits and reduced cerebral infarct volume. In addition, it was observed that the ERK1/2 pathway was activated 24 h after cerebral I/R injury, which was consistent with the findings of previous studies (22,23). However, EA significantly further upregulated ERK1/2 in I/R-injured brain tissues. The pattern of cyclin D1 and CDK4 protein expression was consistent with that of ERK activation in the present study. Consequently, the regulatory effect of EA on ERK activation resulted in the promotion of cerebral cell proliferation.

In conclusion, to the best of our knowledge, the present study reported for the first time that EA at the Quchi (LI11) and Zusanli (ST36) acupoints exerts a neuroprotective effect in ischemic stroke via the activation of the ERK1/2 pathway. These results suggest that EA may be a potential therapeutic approach for the treatment of cerebral ischemia.

## Figures and Tables

**Table I:** Neurological deficit score

Group (n=8)	2 h after I/R	24 h after I/R
SC	0	0
IC	2.50±0.76	2.25±0.71
EA	2.37±0.74	1.50±0.53^a^

Data shown as mean ± SE from 8 individual rats in each group.

a P<0.05, vs. the IC group. SC, sham-operated control; IC, ischemic control; EA, electroacupuncture; I/R, ischemia/reperfusion.

## Abbreviations:

ERK: extracellular signal-regulated kinase;
I/R: ischemia/reperfusion;
MCAO: middle cerebral artery occlusion;
EA: electroacupuncture;
PCNA: proliferating cell nuclear antigen;
TTC: 2,3,5-triphenyltetrazolium chloride;
CDK4: cyclin-dependent kinase 4;
MAPKs: mitogen-activated protein kinases

## References

[1] Luitse MJ, Biessels GJ, Rutten GE, Kappelle LJ (2012). Diabetes, hyperglycaemia, and acute ischaemic stroke. Lancet Neurol.

[2] Szydlowska K, Tymianski M (2010). Calcium, ischemia and excitotoxicity. Cell Calcium.

[3] Wu JN (1996). A short history of acupuncture. J Altern Complement Med.

[4] Kim SK, Bae H (2010). Acupuncture and immune modulation. Auton Neurosci.

[5] Zhang GC, Fu WB, Xu NG (2012). Meta analysis of the curative effect of acupuncture on post-stroke depression. J Tradit Chin Med.

[6] Hu HH, Chung C, Liu TJ (1993). A randomized controlled trial on the treatment for acute partial ischemic stroke with acupuncture. Neuroepidemiology.

[7] Jansen G, Lundeberg T, Kjartansson J, Samuelson UE (1989). Acupuncture and sensory neuropeptides increase cutaneous blood flow in rats. Neurosci Lett.

[8] Johansson K, Lindgren I, Widner H (1993). Can sensory stimulation improve the functional outcome in stroke patients?. Neurology.

[9] Magnusson M, Johansson K, Johansson BB (1994). Sensory stimulation promotes normalization of postural control after stroke. Stroke.

[10] Pavlikova M, Kovalska M, Tatarkova Z (2011). Response of secretory pathways Ca(2+) ATPase gene expression to hyperhomocysteinemia and/or ischemic preconditioning in rat cerebral cortex and hippocampus. Gen Physiol Biophys.

[11] Urban P, Pavlíková M M, Sivonová M (2009). Molecular analysis of endoplasmic reticulum stress response after global forebrain ischemia/reperfusion in rats: effect of neuroprotectant simvastatin. Cell Mol Neurobiol.

[12] Riedemann NC, Ward PA (2003). Complement in ischemia reperfusion injury. Am J Pathol.

[13] Lehotský J, Urban P, Pavlíková M (2009). Molecular mechanisms leading to neuroprotection/ischemic tolerance: effect of preconditioning on the stress reaction of endoplasmic reticulum. Cell Mol Neurobiol.

[14] Seger R, Krebs EG (1995). The MAPK signaling cascade. FASEB J.

[15] Lavoie JN, Rivard N, L’Allemain G, Pouysségur J (1996). A temporal and biochemical link between growth factor-activated MAP kinases, cyclin D1 induction and cell cycle entry. Prog Cell Cycle Res.

[16] Whitmarsh AJ, Davis RJ (2000). A central control for cell growth. Nature.

[17] Gu Z, Jiang Q, Zhang G (2001). Extracellular signal-regulated kinase 1/2 activation in hippocampus after cerebral ischemia may not interfere with postischemic cell death. Brain Res.

[18] Hu BR, Wieloch T (1994). Tyrosine phosphorylation and activation of mitogen-activated protein kinase in the rat brain following transient cerebral ischemia. J Neurochem.

[19] Kurino M, Fukunaga K, Ushio Y, Miyamoto E (1995). Activation of mitogen-activated protein kinase in cultured rat hippocampal neurons by stimulation of glutamate receptors. J Neurochem.

[20] Longa EZ, Weinstein PR, Carlson S, Cummins R (1989). Reversible middle cerebral artery occlusion without craniectomy in rats. Stroke.

[21] Bederson JB, Pitts LH, Germano SM (1986). Evaluation of 2,3,5-triphenyltetrazolium chloride as a stain for detection and quantification of experimental cerebral infarction in rats. Stroke.

[22] Hu X, Wu X, Xu J (2009). Src kinase up-regulates the ERK cascade through inactivation of protein phosphatase 2A following cerebral ischemia. BMC Neurosci.

[23] Sugino T, Nozaki K, Takagi Y (2000). Activation of mitogen-activated protein kinases after transient forebrain ischemia in gerbil hippocampus. J Neurosci.

[24] Boulton TG, Nye SH, Robbins DJ (1991). ERKs: a family of protein-serine/threonine kinases that are activated and tyrosine phosphorylated in response to insulin and NGF. Cell.

[25] Nishida E, Gotoh Y (1993). The MAP kinase cascade is essential for diverse signal transduction pathways. Trends Biochem Sci.

[26] Aikawa R, Komuro I, Yamazaki T (1997). Oxidative stress activates extracellular signal-regulated kinases through Src and Ras in cultured cardiac myocytes of neonatal rats. J Clin Invest.

[27] Fiore RS, Murphy TH, Sanghera JS (1993). Activation of p42 mitogen-activated protein kinase by glutamate receptor stimulation in rat primary cortical cultures. J Neurochem.

[28] Jirmanova L, Afanassieff M, Gobert-Gosse S (2002). Differential contributions of ERK and PI3-kinase to the regulation of cyclin D1 expression and to the control of the G1/S transition in mouse embryonic stem cells. Oncogene.

[29] Tian HP, Huang BS, Zhao J (2009). Non-receptor tyrosine kinase Src is required for ischemia-stimulated neuronal cell proliferation via Raf/ERK/CREB activation in the dentate gyrus. BMC Neurosci.

[30] Zhou L, Miller CA (2006). Mitogen-activated protein kinase signaling, oxygen sensors and hypoxic induction of neurogenesis. Neurodegener Dis.

[31] Zhou L, Del Villar K, Dong Z, Miller CA (2004). Neurogenesis response to hypoxia-induced cell death: map kinase signal transduction mechanisms. Brain Res.

[32] Luo WS, Yu HB, Yang ZX (2010). Influence of Ren and Du meridian electro-acupuncture on neural stem cell proliferation and extracellular signal-regulated kinase pathway in a rat model of focal cerebral ischemia injury. Neural Regen Res.

